# Phosphorylation of eIF2α signaling pathway attenuates obesity-induced non-alcoholic fatty liver disease in an ER stress and autophagy-dependent manner

**DOI:** 10.1038/s41419-020-03264-5

**Published:** 2020-12-14

**Authors:** Jie Li, Xinle Li, Daquan Liu, Shiqi Zhang, Nian Tan, Hiroki Yokota, Ping Zhang

**Affiliations:** 1 Department of Anatomy and Histology, School of Basic Medical Sciences, Tianjin Medical University, Tianjin, 300070 China; 2 Key Laboratory of Hormones and Development (Ministry of Health), Tianjin Key Laboratory of Metabolic Diseases, Tianjin Medical University, Tianjin, 300070 China; 3 Department of Biomedical Engineering, Indiana University-Purdue University, Indianapolis, IN 46202 USA; 4 Tianjin Key Laboratory of Spine and Spinal Cord, Tianjin Medical University, Tianjin, 300052 China

**Keywords:** Obesity, Metabolic syndrome, Outcomes research

## Abstract

Non-alcoholic fatty liver disease (NAFLD) is the most common liver disorder and frequently exacerbates in postmenopausal women. In NAFLD, the endoplasmic reticulum (ER) plays an important role in lipid metabolism, in which salubrinal is a selective inhibitor of eIF2α de-phosphorylation in response to ER stress. To determine the potential mechanism of obesity-induced NAFLD, we employed salubrinal and evaluated the effect of ER stress and autophagy on lipid metabolism. Ninety-five female C57BL/6 mice were randomly divided into five groups: standard chow diet, high-fat (HF) diet, HF with salubrinal, HF with ovariectomy, and HF with ovariectomy and salubrinal. All mice except for SC were given HF diet. After the 8-week obesity induction, salubrinal was subcutaneously injected for the next 8 weeks. The expression of ER stress and autophagy markers was evaluated in vivo and in vitro. Compared to the normal mice, the serum lipid level and adipose tissue were increased in obese mice, while salubrinal attenuated obesity by blocking lipid disorder. Also, the histological severity of hepatic steatosis and fibrosis in the liver and lipidosis was suppressed in response to salubrinal. Furthermore, salubrinal inhibited ER stress by increasing the expression of p-eIF2α and ATF4 with a decrease in the level of CHOP. It promoted autophagy by increasing LC3II/I and inhibiting p62. Correlation analysis indicated that lipogenesis in the development of NAFLD was associated with ER stress. Collectively, we demonstrated that eIF2α played a key role in obesity-induced NAFLD, and salubrinal alleviated hepatic steatosis and lipid metabolism by altering ER stress and autophagy through eIF2α signaling.

## Introduction

Non-alcoholic fatty liver disease (NAFLD) is a syndrome, characterized by intrahepatic lipid deposition in the absence of causes such as alcohol, viruses, and drugs^1^. The average prevalence of NAFLD is 20–30%^2,3^. The incidence of NAFLD may be linked with obesity, high-fat diet (HFD), and postmenopausal^4,5^. These factors are likely to increase body fat, and hormone disorders may interact with obesity-linked metabolic changes, which make identification of its pathogenic causes difficult. Previous studies indicated that HFD in combination with ovariectomy-induced obesity causes lipid metabolism disorders as well as liver steatosis^6^. However, the pathogenesis of obesity-induced hepatic steatosis, particularly in obesity and postmenopausal women, has not been elucidated.

Several factors, such as endoplasmic reticulum (ER) stress, oxidative stress, and insulin resistance, are known to influence the pathogenesis of NAFLD^7^. It is reported that ER stress is linked to HFD-driven steatosis^8^. Stress to the ER response is the cell’s self-defense mechanism, which is mainly mediated by three transmembrane receptor proteins, such as inositol requires kinase 1 (IRE1), double-stranded RNA-activated protein kinase (PKR)-like ER kinase (PERK), and activated transcription factor 6 (ATF6)^9^. Pre-clinical studies showed that carbon monoxide alleviated methionine/choline deficient diet-induced hepatic steatosis, by up-regulating sestrin-2 via the PERK/eIF2α/ATF4 signaling pathway^10^. While steatosis is observed in response to ER stress, recent studies have indicated that ER stress directly plays a crucial role in the regulation of lipid metabolism. The study suggested that activation of the PERK/p-eIF2α signaling pathway by antipsychotic drugs (APDs) increased intracellular lipid accumulation via activation of SREBP-1c and SREBP-2 in hepatocytes^11,12^. In addition, enforced expression of GADD34 (eIF2α specific phosphatase) markedly altered the metabolic profile and reduced a high-fat diet-induced hepatic lipid deposition in GADD34 transgenic mice^13^. Although the ER stress has been reported to be linked to fatty liver, the key role of ER stress in the obesity-induced NAFLD remains unclear.

Autophagy is an essential physiological process to maintain the transformation of intracellular substances, and ER is suggested to be one of the membrane sources during autophagic vesicle formation^14^. Autophagy induced by ER stress may have involved in the process of lipophagy through selective lipid droplets^15^. In vivo and in vitro experiments indicated that a decrease in intracellular lipid accumulation and alleviation of hepatic steatosis in autophagy might be a potential strategy to treat NAFLD^16,17^. However, the contribution of ER stress and autophagy to NAFLD is yet to be clarified.

Salubrinal is a selective inhibitor of the de-phosphorylation of eIF2α, which maintains a phosphorylation status of eIF2α and protects cells from ER stress-induced apoptosis^18^. Studies have shown that salubrinal improves pancreatitis, neurodegenerative diseases, and provides therapeutic efficacy in metabolic diseases such as leptin sensitivity^19–21^. Our previous studies showed that salubrinal promotes the healing of surgical wounds by maintaining phosphorylation of eIF2α in rodent models^22^. Moreover, salubrinal stimulated angiogenesis and bone formation and improve ischemic osteonecrosis^23^. In addition, salubrinal prevented bone loss and regulating bone marrow mesenchymal stem cells to differentiate into osteoblasts^24,25^. Our recent study demonstrates that salubrinal attenuates bone loss by regulating ER stress-autophagy axis through promoting the expression of LC3II/I of osteoblasts and altering the proliferation and differentiation of osteoclasts by regulating eIF2α^26^. Although salubrinal has an effect on many diseases through the eIF2α signaling pathway, little is known about its role in obesity and lipid metabolism.

Herein, we hypothesized that ER stress triggers the progression of obesity-induced NAFLD with hepatic steatosis and lipidosis, and salubrinal restored lipid metabolism by inhibiting ER stress and promoting autophagy via the eIF2α signaling pathway. To test the hypothesis, an obesity model induced by a high-fat diet or combined with ovariectomy was employed. Salubrinal was subcutaneously injected, and we evaluated the effect of salubrinal on lipid metabolism using biochemical analysis, histology, and cytology. In particular, we examined the inhibitory mechanism of lipid deposition in hepatocytes by salubrinal in vivo and in vitro.

## Materials and methods

### Animals and material preparation

Ninety-five female C57BL/6 mice (Animal Center of Academy of Military Medical Sciences, China), ~14-week of age were used. The mice were maintained at 25 °C in a 12 h light-dark cycle under pathogen-free conditions and had free access to water and food. All experiments were carried out according to the National Institutes of Health Guide for Care and Use of Laboratory Animals and were approved by the Ethics Committee of Tianjin Medical University.

The salubrinal was purchased from Tocris Bioscience (Ellisville, MO, USA). Primary antibodies of eIF2α, phospho-eIF2α, ATF4 were purchased from Cell Signaling (Danvers, MA, USA). CHOP was purchased from Proteintech (Wuhan, Hubei, China). LC3 and p62 were purchased from MBL (Co, Nagoya, Aichi-ken, Japan). MEM-α, fetal bovine serum, penicillin, streptomycin, and trypsin were purchased from Invitrogen (Waltham, MA, USA). Other chemicals were purchased from Sigma (St. Louis, MO, USA) unless otherwise stated^24^.

### Experimental design

**Fig. 1:**
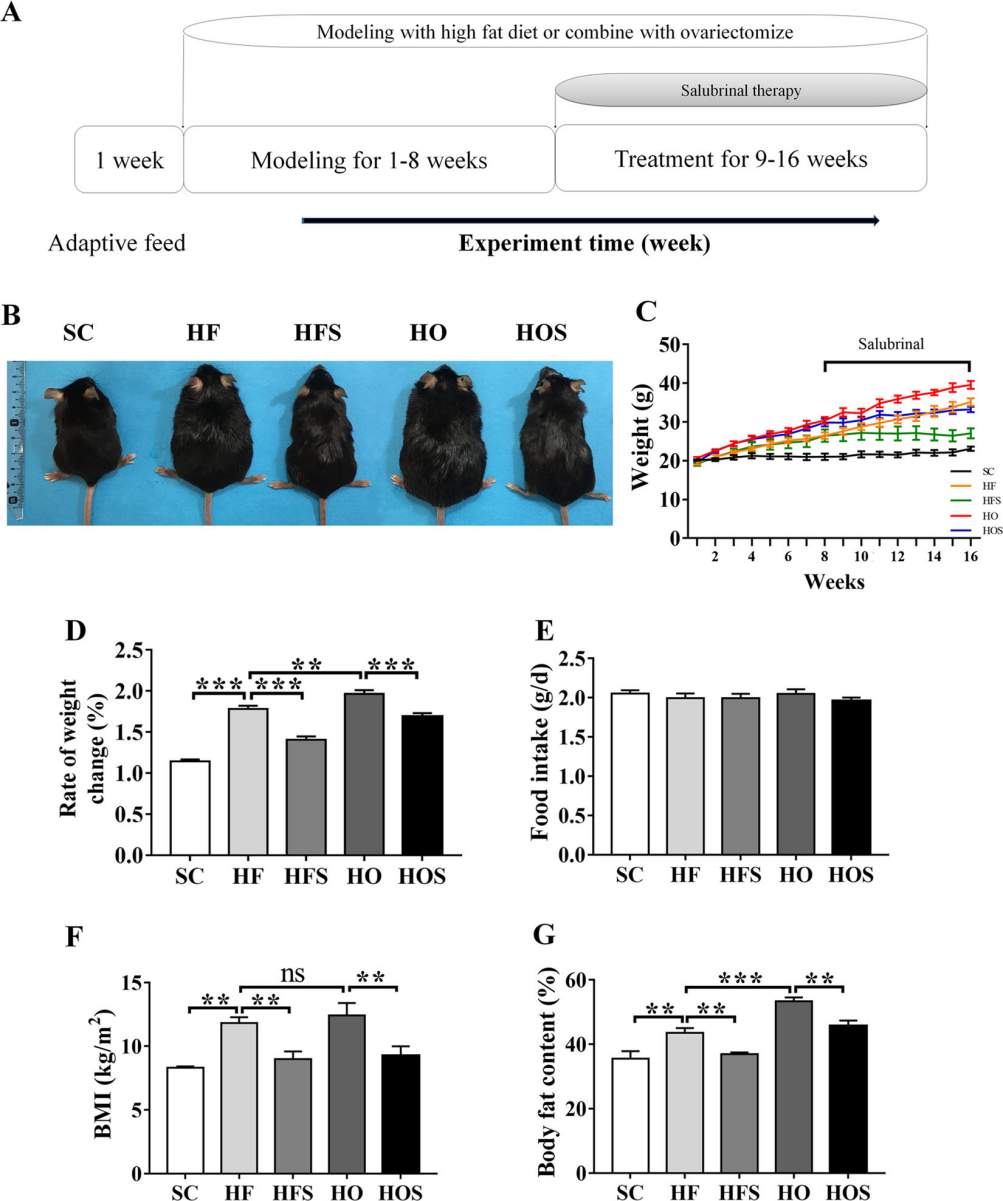
Salubrinal attenuated obesity. **a** Experiment timeline. **b** Whole Body image. **c** Weight change. **d** Rate of weight change. **e** Daily food intake (per day among five groups). **f** Body mass index (BMI). **g** Whole-body fat content. All values were reported as mean ± SEM (*n* = 10/groups and images were taken randomly). The asterisks (*, **, and ***) represent *P* < 0.05, *P* < 0.01, and *P* < 0.001, respectively. N.s., not significant.

After 1-week acclimation, mice were randomly divided into five groups: standard chow diet (SC; *n* = 19), high-fat diet group (HF; *n* = 19, D12492, Beijing Huafu Kang Biological Co, Beijing, China), high-fat diet with salubrinal (HFS; *n* = 19), high-fat diet in a combination of ovariectomy (HO; *n* = 19), and a high-fat diet in a combination of ovariectomy with salubrinal (HOS; *n* = 19). Two OVX groups (HO and HOS) underwent ovariectomy, while three sham OVX groups (SC, HF, and HFS) were subjected to sham surgery. All mice except for SC were fed with a high-fat diet. After 8 weeks of a high-fat diet, two groups (HFS and HO) received a subcutaneous injection of salubrinal for 8 weeks (salubrinal injection dose: 1 mg/kg; Fig. 1a)^6^.

### Ovariectomy

Mice were anesthetized with 1.5% isoflurane (IsoFlo: Abbott Laboratories, North Chicago, USA) at a flow rate of 1.0 L/min. The mice were placed in a prone position after being comatose. Ovariectomy was performed via a midline dorsal incision under sterile conditions after which both ovaries were identified adjacent to the inferior pole of the kidneys in the peritoneal cavity. The bilateral ovaries were removed and the tissue layers were closed and sutured^27^.

### Body fat composition and biochemical analysis

The food intake was evaluated daily, and the body-weight was measured weekly, by a person blinded to each group. Body fat content was measured by a whole-body composition analyzer (ImpediVet, Pinkenba, Qld, Australia). Blood samples were obtained after fasted and water deprivation for 12 h. After fasting, the blood glucose and serum levels of triglycerides and total cholesterol were measured by colorimetric kit (CardioChek, Indianapolis, IN, USA). The concentration of the insulin level was assessed by enzyme-linked immunosorbent assays (Cloud-Clone Corp, Houston, TX, USA)^6^.

### Histological analysis

Liver and periuterine fat samples were fixed in 10% formaldehyde for 48 h and embedded in paraffin or used for frozen slices^6^. To analyze hepatic steatosis, liver sections were stained with H&E and oil red O staining, and the fat vacuole area and quantity were measured. Liver steatosis was examined by histopathology and classified as: grade 0, 1–5% coverage; grade 1, 6–33% coverage; grade 2, 34–66% coverage; and grade 3, 67–100% coverage^28^. To examine liver fibrosis, liver sections were processed with Masson’s trichrome staining. All graphics were taken at random and measured using the Cellsense Standard software for measurement assessment^29^.

### Induction of hepatocyte steatosis and adipogenic development

HepG2 and 3T3-L1 cells were cultured in α-MEM supplemented with 10% fetal bovine serum. At 80% confluency, HepG2 cells were treated with 12 mM oleic acid (OA) or supplemented with 10 μM salubrinal for 2 days^30^. To induce adipogenic differentiation, 50 μM indomethacin, 0.5 μM IBMX, 0.5 μM dexamethasone, and 5 μg/ml insulin were added to the complete medium. 3T3-L1 cells were treated with 1 μM tunicamycin (TM), 1 ng/ml bafilomycin A1 (BA1), or 10 μM salubrinal. After incubation for 3 days, the complete medium containing only 5 μg/ml insulin was replaced^31^.

### Cell oil red O staining

Fully differentiated adipocytes were fixed with 4% formaldehyde fixative solution and stained with 60% oil red O dye solution. To examine lipid accumulation, 100% isopropanol was added to extract oil red and determined at the absorbance of 520 nm^32^.

### Immunofluorescence analysis of LC3

Immunofluorescence analysis was conducted as described previously^26,33^. 3T3-L1 cells were cultured with DMSO (vehicle), 5 μM salubrinal, 100 ng/ml tunicamycin, or 1 ng/ml bafilomycin A1 for 48 h. They were incubated with LC3 antibody for labeling autophagosomes, followed by incubation with fluorescent antibody in dark. DAPI was employed to counterstain the nuclei. The experiment was conducted in triplicate.

### Western blot analysis

For western blot analysis, we isolated proteins from the liver, HepG2 cells, and 3T3-L1 cells. Specimens were lysed in a RIPA lysis buffer, containing protease inhibitors and phosphatase inhibitors. Isolated proteins were fractionated using 10% sodium dodecyl sulfate-polyacrylamide gels and electrotransferred to polyvinylidene difluoride membranes. Primary antibodies specific to collagen IV, eIF2α, phospho-eIF2α, ATF4, CHOP, p62, LC3, and β-actin were employed. After incubation with secondary lgG antibodies incubated again with horseradish peroxidase, signals were detected by enhanced chemiluminescence. Data were analyzed and quantified with reference to the intensities of β-actin^34^. The experiments were conducted in triplicate.

### Statistical analysis

The data were presented as mean ± SEM (standard error). One-way ANOVA was employed to determine significant differences. Correlation analysis was performed using the Pearson correlation coefficient test. All comparisons were two-tailed and statistical significance was assumed at *P* < 0.05. The asterisks (*, **, and ***) represent *P* < 0.05, *P* < 0.01, and *P* < 0.001, respectively.

## Results

### Salubrinal attenuated obesity

The mean body weight and its rate of change were determined every week. Compared to SC, a significant weight increase was observed in HF and HO (both *P* < 0.001). Moreover, HO had a higher body weight change than HF (*P* < 0.01). In response to salubrinal, however, HFS reduced body weight, and HOS showed a lower body weight than HO (both *P* < 0.001; Fig. 1b–d). Of note, food intake did no statistical difference among groups (Fig. 1e).

To analyze the changes in body fat of each group, BMI index, and body fat content were evaluated using whole-body composition. HF and HO showed significantly higher body fat than SC in a high-fat diet (both *P* < 0.01). However, salubrinal markedly reduced body fat in HFS and HOS (both *P* < 0.01; Fig. 1f, g).

### Salubrinal reduced the mass of adipocyte tissue

**Fig. 2:**
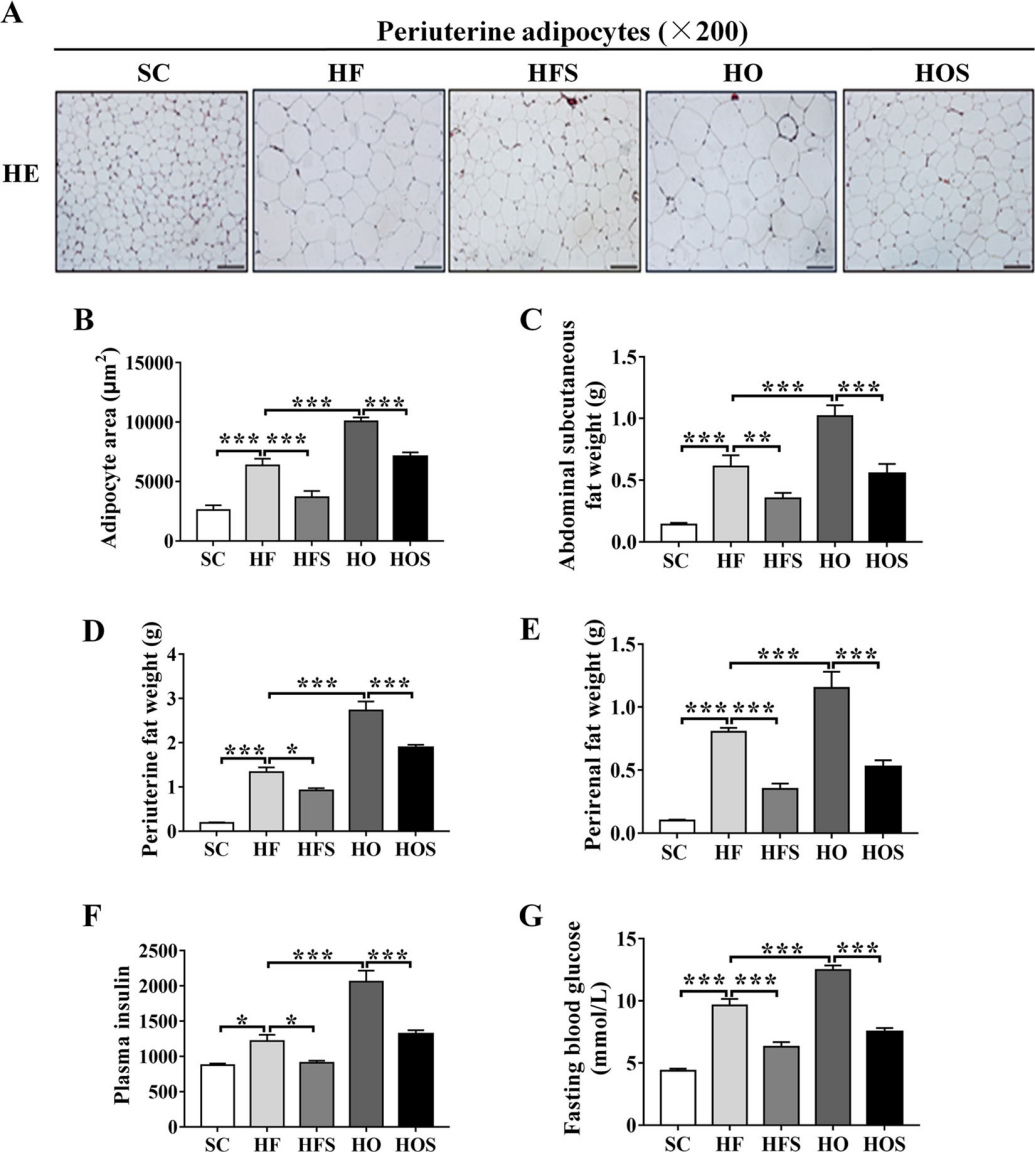
Salubrinal reduced adipocyte tissue mass. **a** Representative H&E staining images showing salubrinal effect in periuterine tissue (200×; scale bar = 100 μm; all graphics were in a blinded fashion). **b** Area of adipocytes. **c** Abdominal subcutaneous fat weight. **d** Periuterine fat weight. **e** Perirenal fat weight. **f** Serum insulin. **g** Fasting glucose. All values were reported as mean ± SEM (*n* = 10/groups and images were taken randomly). The asterisks (*, **, and ***) represent *P* < 0.05, *P* < 0.01, and *P* < 0.001, respectively.

To examine the hypertrophic change, the adipocyte area was evaluated using H&E stained periuterine adipose tissues. Compared to SC, histologic examination showed that adipocytes were larger in HF and HO (both *P* < 0.001), and HO had a significant increase than HF (*P* < 0.001). In response to salubrinal, the hypertrophic change of adipocytes in the two obese groups was suppressed (both *P* < 0.001; Fig. 2a, b). To determine the effect of salubrinal on lipid metabolism, we evaluated the weight of abdominal subcutaneous fat, periuterine, and perirenal fat tissues. Compared to SC, the adipose tissue of HF was increased with a high-fat diet (both *P* < 0.001), and fat accumulation was a significant increase in HO (both *P* < 0.001). In contrast, the adipose tissues were significantly decreased in HFS and HOS after salubrinal injected for 8-week (both *P* < 0.05; Fig. 2c–e).

Furthermore, the levels of plasma insulin and fasting blood glucose were used to examine insulin resistance. Compared to SC, the plasma insulin and fasting blood glucose levels in HF and HO were significantly increased (both *P* < 0.05). However, HFS improved insulin resistance compared with HF (*P* < 0.05), and HOS showed a drastic reduction of plasma insulin and fasting blood glucose levels in HO (both *P* < 0.001; Fig. 2f, g).

### Salubrinal alleviated obesity-induced hepatic steatosis

**Fig. 3:**
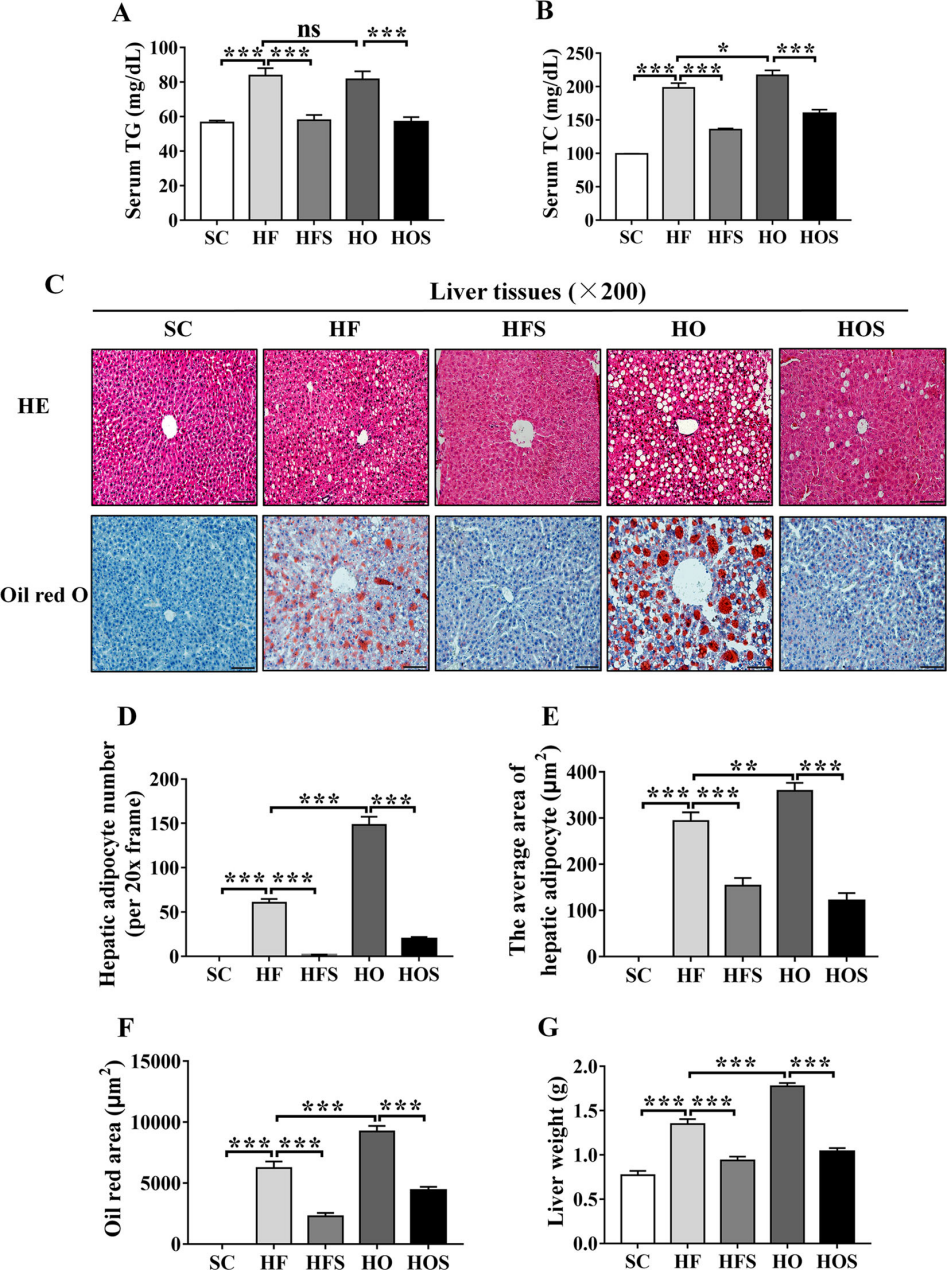
Salubrinal improved obesity-induced hepatic steatosis. **a** Serum triglyceride level. **b** Serum total cholesterol level. **c** Representative H&E and oil red O staining images showing salubrinal effect in hepatic steatosis (200×; scale bar = 100 μm; all graphics were in a blinded fashion). **d** The number of hepatic adipocytes. **e** The average area of hepatic adipocyte. **f** Oil red area. **g** Liver weight. The asterisks (*, **, and ***) represent *P* < 0.05, *P* < 0.01, and *P* < 0.001, respectively. N.s., not significant.

The serum triglyceride and total cholesterol were used to identify hyperlipidemia. Compared to SC, the serum lipid level was significantly increased in HF and HO (both *P* < 0.001). However, HFS decreased the high-level of serum triglyceride and total cholesterol in HF (both *P* < 0.001), and serum lipid level in HOS was decreased compared with the HO (both *P* < 0.001; Fig. 3a, b). To determine the hepatic steatosis, we evaluated lipid droplets number and area using H&E staining and oil red O staining with hepatic sections. Compared to SC, the excessive lipid droplets in the cytoplasmic of hepatocytes was observed in HF and HO (both *P* < 0.001), and the degeneration of ballooning hepatocytes was exacerbated in HO compared to HF (*P* < 0.01). In response to salubrinal, HFS and HOS significantly attenuated ballooning hepatocytes compared with the obese groups (both *P* < 0.001; Fig. 3c–f). In addition, compared to SC, liver weight was significantly increased in HF, and it was further increased in HO (both *P* < 0.001). However, the increase in liver weight in obese groups was suppressed by salubrinal (both *P* < 0.001; Fig. 3g).

### Salubrinal improved obesity-induced hepatic fibrosis

**Fig. 4:**
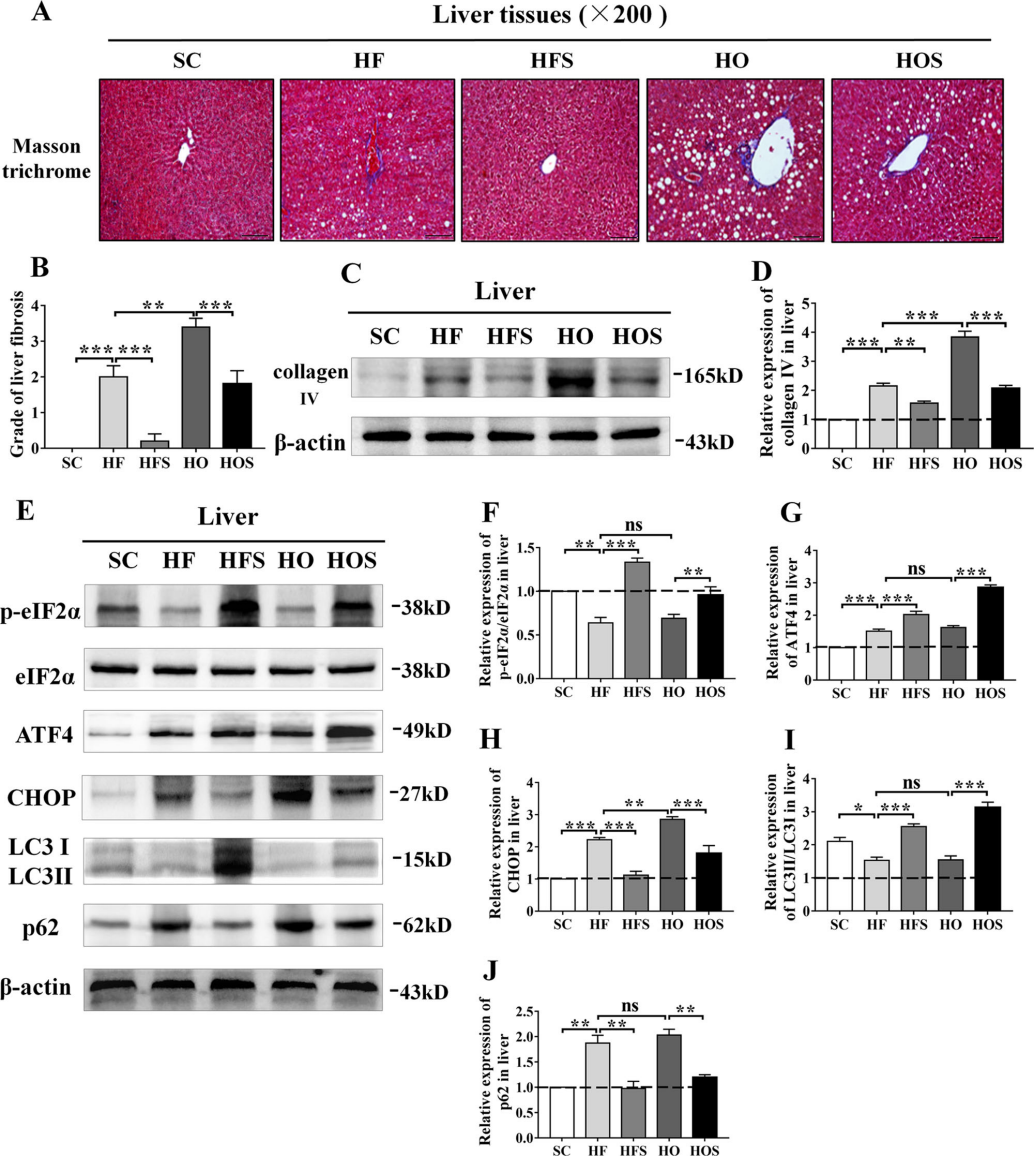
Salubrinal alleviated liver fibrosis and altered the ER stress and autophagy in vivo. **a** Representative Masson-trichrome staining images showing salubrinal effect in liver fibrosis (200×; scale bar = 100 μm; all graphics were in a blinded fashion). **b** Grade of liver fibrosis. **c** Representative images of Western blot for collagen IV. **d** Relative intensity of collagen IV. **e** Representative images of Western blot in the five groups for selected genes involved in ER stress (p-eIF2α, ATF4, and CHOP) and autophagy (LC3 and p62). **f–j** Relative intensity of p-eIF2α/eIF2α (**f**), ATF4 (**g**), CHOP (**h**), LC3II/I (**i**), p62 (**j**). The experiments were conducted in triplicate. The asterisks (*, **, and ***) represent *P* < 0.05, *P* < 0.01, and *P* < 0.001, respectively. N.s., not significant.

To further investigate liver fibrosis, we analyzed liver sections using Masson’s trichrome staining. Compared to SC, mild fibrosis was detected in HF (*P* < 0.001), and fibrosis in HO was aggravated compared with the HF (*P* < 0.01). However, HFS reduced the grading of liver fibrosis (*P* < 0.001), and HOS showed a significant decrease compared to HO (*P* < 0.001; Fig. 4a, b). The expression of collagen IV was performed in a similar fiberization trend. Compared with the SC, the expression of collagen IV was remarkably increased in HF and HO (both *P* < 0.001). In response to salubrinal, HFS decreased the level of collagen IV (*P* < 0.01) and HOS showed a significant decrease compared to HO (*P* < 0.001; Fig. 4c, d).

### Salubrinal inhibited hepatic ER stress and promoted autophagy by maintaining eIF2α phosphorylation

To investigate ER stress and autophagy in response to salubrinal on hepatic steatosis, the levels of biomarkers involved in ER stress and autophagy were evaluated (Fig. 4e). Compared to SC, the expression of p-eIF2α/eIF2α was significantly decreased (both *P* < 0.01), and ATF4 and CHOP were increased in HF and HO (both *P* < 0.001). However, salubrinal restored the level of p-eIF2α/eIF2α (*P* < 0.01) and further increased the level of ATF4 (both *P* < 0.001), while inhibited the level of CHOP in obese groups (both *P* < 0.001; Fig. 4f–h). Compared to SC, the expression of LC3II/I was decreased in HF and HO (both *P* < 0.05). However, the results showed that the level of LC3II/I in HFS and HOS was significantly increased than that in obese mice (both *P* < 0.001; Fig. 4i). The expression of p62 in HF and HO was increased than that in SC (both *P* < 0.01). In response to salubrinal, HFS decreased the level of p62 compared to the HF, and HOS showed its significant decrease than that in HO (both *P* < 0.01; Fig. 4j).

### Salubrinal suppressed fatty degeneration against the ER stress and promoted autophagy by maintaining eIF2α phosphorylation in HepG2 cells

**Fig. 5:**
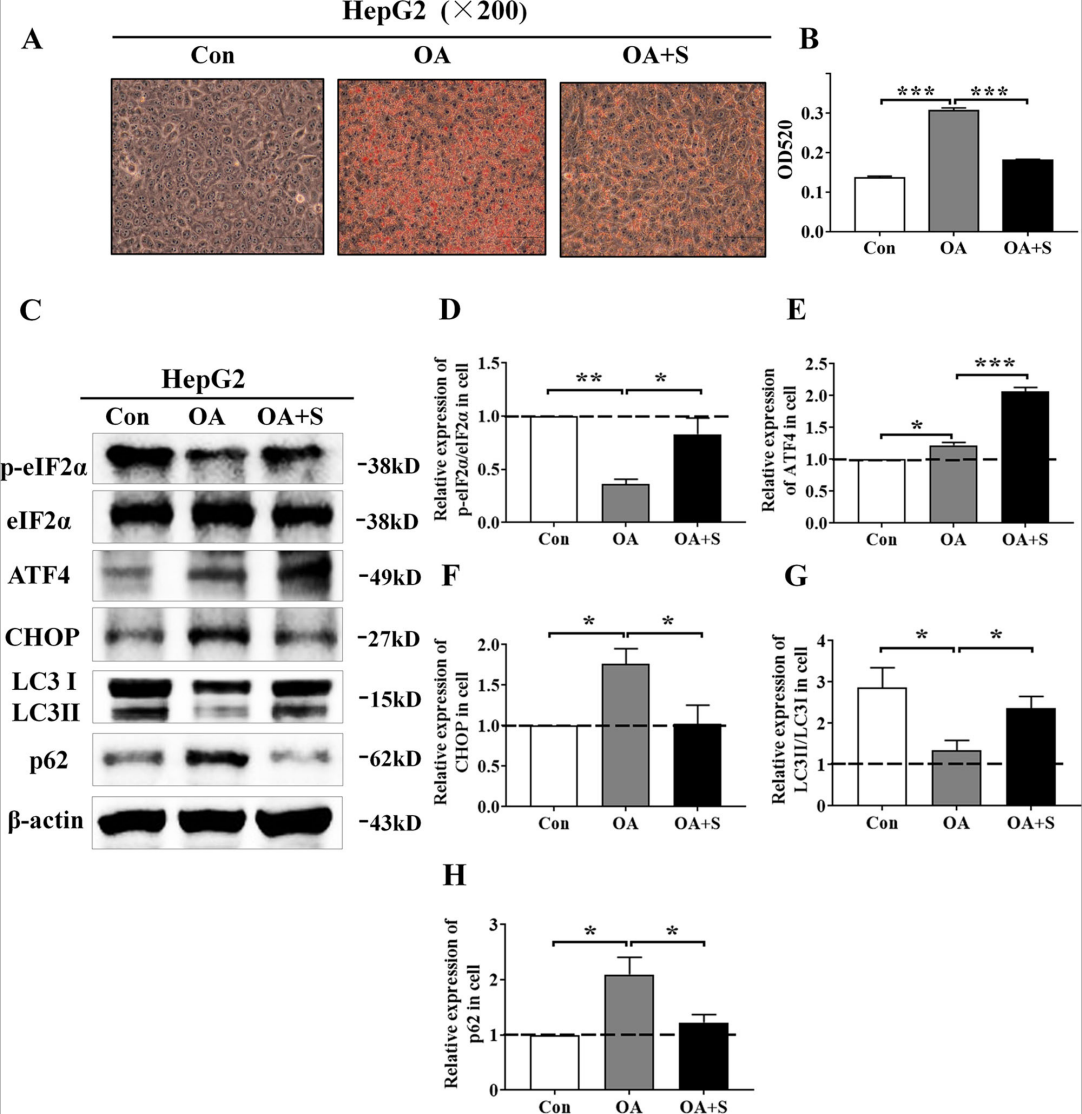
Salubrinal impeded fatty degeneration in HepG2 cells. **a, b** Representative oil red O staining images showing salubrinal effects in HepG2 cells and quantitative analysis by OD_520_ (200×; scale bar = 100 μm; all graphics were processed in a blinded fashion). **c** Representative images of Western blot for the selected genes involved in the ER stress in HepG2 cells (p-eIF2α, ATF4, and CHOP) and autophagy (LC3 and p62). **d–h** Relative intensities of p-eIF2α/eIF2α (**d**), ATF4 (**e**), CHOP (**f**), LC3II/I (**g**), and p62 (**h**). The experiments were conducted in triplicate. The asterisks (*, **, and ***) represent *P* < 0.05, *P* < 0.01, and *P* < 0.001, respectively (S: salubrinal and OA: oleic acid).

The oleic acid (OA) was used as an inducer of fatty degeneration in hepatocytes. Compared to the vehicle group, the hepatic steatosis and lipid droplets were dramatically increased in the oleic acid-inducing group (*P* < 0.001), whereas the oil red O staining showed that salubrinal alleviated fatty degeneration and inhibited lipid deposition in HepG2 cells (*P* < 0.001; Fig. 5a, b).

To further investigate the effect of ER stress on lipidosis in response to salubrinal, the levels of biomarkers in HepG2 cells involved in the ER stress and autophagy were evaluated (Fig. 5c). Western blot analysis demonstrated that salubrinal increased the level of p-eIF2α/eIF2α (*P* < 0.05) and ATF4 (*P* < 0.001), but decreased the level of CHOP (*P* < 0.05) in HepG2 cells (Fig. 5d–f). Meanwhile, the expression of LC3II/I by oleic acid (OA) was decreased compared to the control (*P* < 0.05), but this level was increased by salubrinal (*P* < 0.05; Fig. 5g). In addition, the expression of p62 by OA was increased than control (*P* < 0.05), but salubrinal decreased the expression of p62 compared with the OA group (*P* < 0.05; Fig. 5h).

### Salubrinal impeded lipidosis against the ER stress and promoted autophagy by maintaining eIF2α phosphorylation in 3T3-L1 cells

**Fig. 6:**
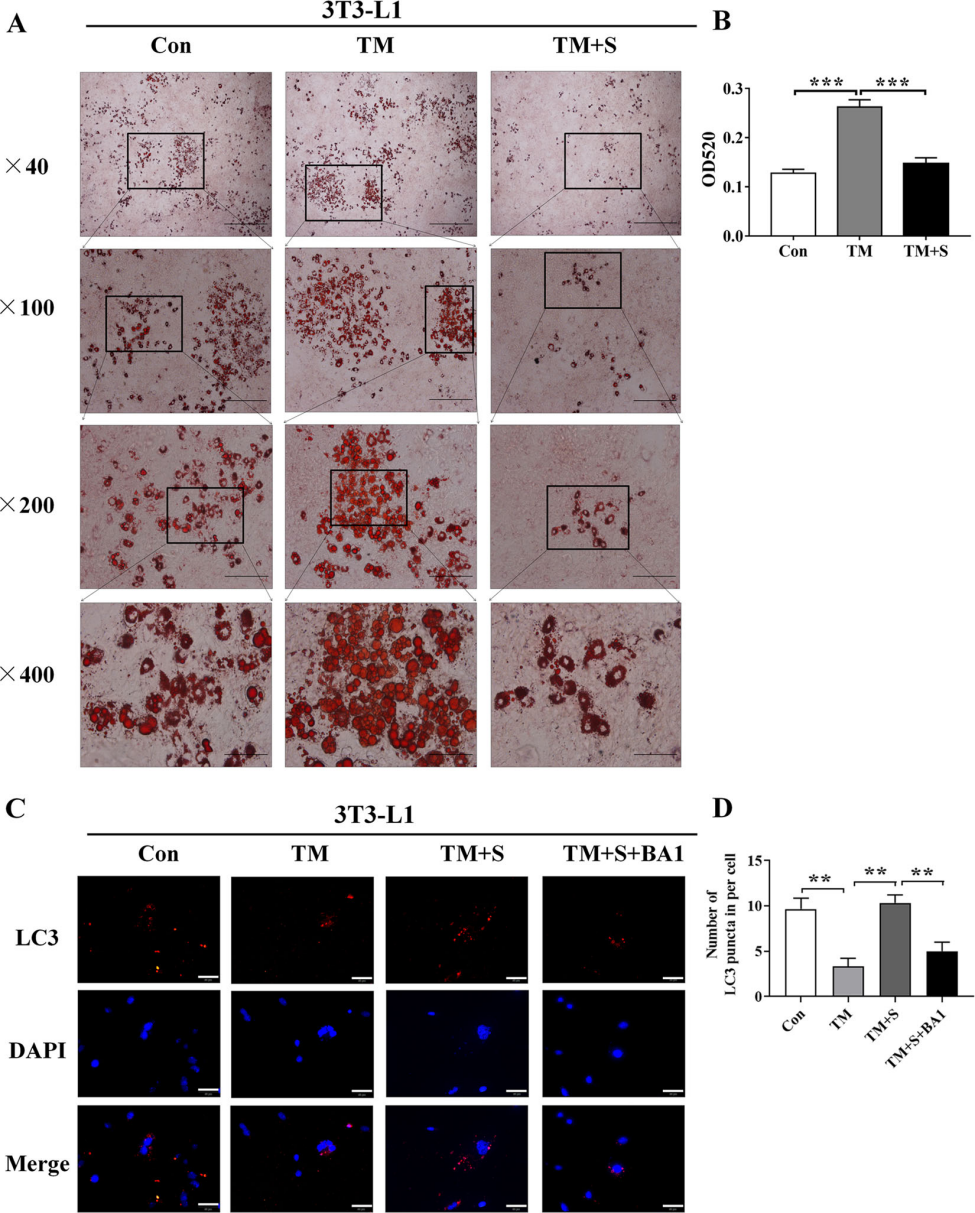
Salubrinal impeded lipidosis in 3T3-L1 cells. **a**, **b** Representative oil red O staining images showing salubrinal effects in 3T3-L1 cells at different magnifications and quantitative analysis by OD_520_ (magnification: 40×, scale bar = 500 μm; 100×, scale bar = 200 μm; 200×, scale bar = 100 μm; 400×, scale bar = 50 μm; all graphics were in a blinded fashion). **c** Representative immunofluorescence images of LC3 in 3T3-L1 cells from the four groups (blue: DAPI; red: LC3^+^ cells; Tm: tunicamycin; Tm + Sal: tunicamycin + salubrinal; Tm + S + BA1: tunicamycin + salubrinal + bafilomycin A1; 400×, Bar = 50 μm). **d** Quantification of LC3 puncta per cell was shown. The experiments were conducted in triplicate. The asterisks (*, **, and ***) represent *P* < 0.05, *P* < 0.01, and *P* < 0.001, respectively.

To evaluate the effects of salubrinal on lipidosis, tunicamycin (TM) was used as an inducer of ER stress to promote differentiation in 3T3-L1 cells. Compared with the vehicle group, adipocyte differentiation was significantly increased in the tunicamycin-inducing group (*P* < 0.001), whereas salubrinal markedly inhibited lipid droplet formation in 3T3-L1 cells (*P* < 0.001; Fig. 6a, b).

To evaluate the mechanism by which salubrinal regulates autophagy in lipidosis, we employed tunicamycin (ER stress inducer), salubrinal, and bafilomycin A1 (autophagy inhibitor), and detected the level of an autophagy marker protein, LC3. Compared to the control group, the number of LC3 puncta was decreased after treatment with tunicamycin, while the autophagic puncta were significantly increased after salubrinal treatment. However, bafilomycin A1 decreased LC3 (Fig. 6c, d).

**Fig. 7:**
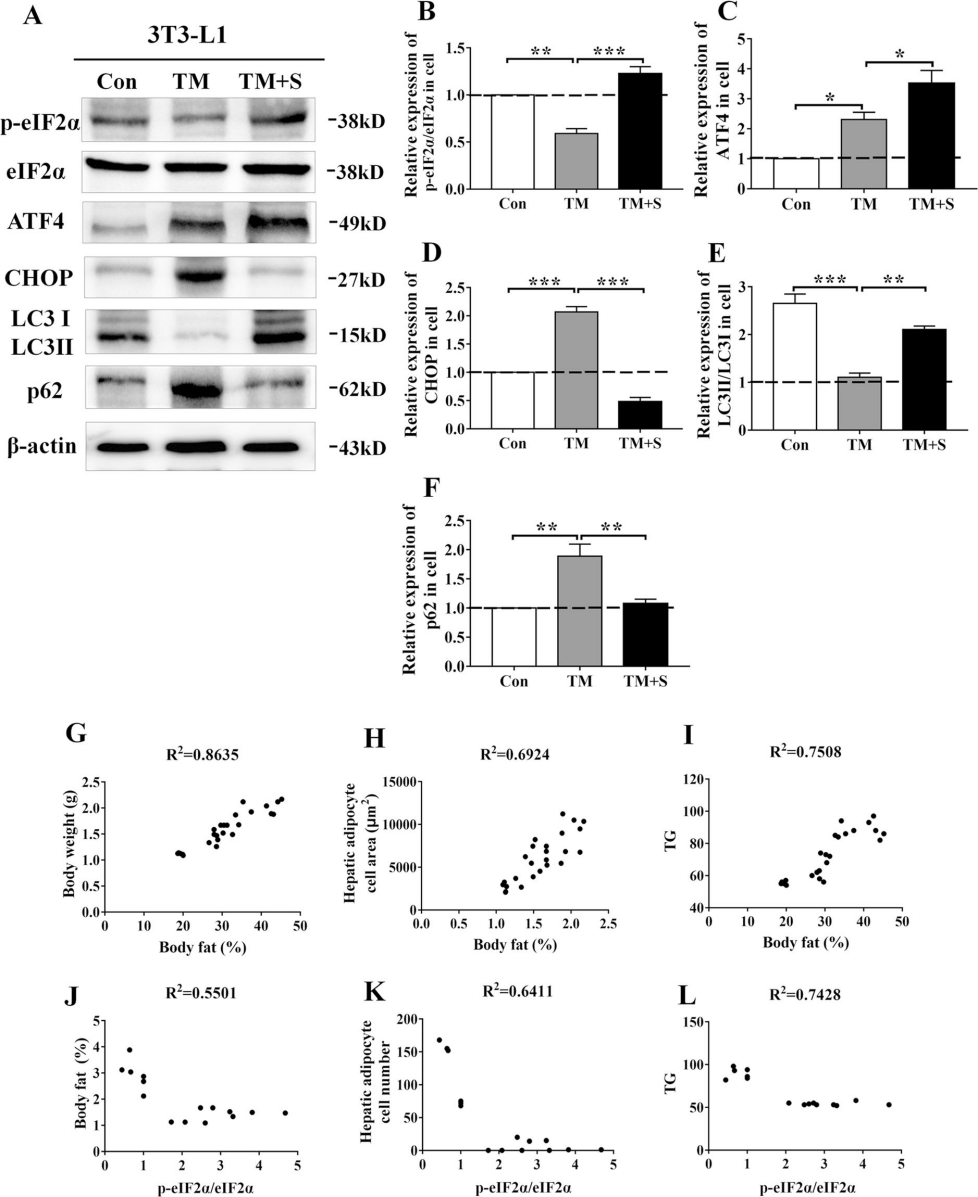
Maintaining eIF2α phosphorylation altered the ER stress and autophagy in 3T3-L1 cells. **a** Representative images of Western blot for selected genes involved in ER stress (p-eIF2α, ATF4, and CHOP) and autophagy (LC3 and p62). **b–f** Relative intensity of p-eIF2α/eIF2α (**b**), ATF4 (**c**), CHOP (**d**), LC3II/I (**e**), p62 (**f**). The experiments were conducted in triplicate. The asterisks (*, **, and ***) represent *P* < 0.05, *P* < 0.01, and *P* < 0.001, respectively (S: salubrinal and TM: tunicamycin). **g–i** The expression level of body fat was positively associated with body weight (**g**), hepatic adipose cell area (**h**), and serum triglyceride (**i**). **j–l** The expression level of p-eIF2α/eIF2α was negatively associated with body fat (**j**), hepatic adipose cell area (**k**), and serum triglyceride (**l**).

To further investigate the effect of ER stress on lipidosis in response to salubrinal, the levels of biomarkers in 3T3-L1 cells involved in ER stress and autophagy were evaluated (Fig. 7a). Western blot analysis demonstrated that salubrinal increased the level of p-eIF2α/eIF2α (*P* < 0.001) and ATF4 (*P* < 0.05) but decreased the level of CHOP (*P* < 0.001) in 3T3-L1 cells (Fig. 7b–d). Meanwhile, the expression of LC3II/I by tunicamycin-inducing was decreased compared with control (*P* < 0.001), this level was increased by salubrinal (*P* < 0.01; Fig. 7e). In addition, the expression of p62 by tunicamycin-inducing was increased than control (*P* < 0.01), and salubrinal decreased the expression of p62 compared with the tunicamycin-inducing group (*P* < 0.01; Fig. 7f).

### The correlations between body fat, p-eIF2α/eIF2α, and lipid metabolism in obesity-induced non-alcoholic fatty liver

To evaluate the effect of salubrinal on hepatic steatosis the correlations between body fat, p-eIF2α/eIF2α and lipid metabolism were analyzed. The body fat was positively associated with body weight (*R*^2^ = 0.8635, *P* < 0.01; Fig. 7g), hepatic adipose cell area (R^2^ = 0.6924, *P* < 0.01; Fig. 7h), and serum triglyceride (*R*^2^ = 0.7508, *P* < 0.01; Fig. 7i). The expression level of p-eIF2α/eIF2α was negatively associated with the body fat (R^2^ = 0.5501, *P* < 0.01; Fig. 7j), hepatic adipocyte number (*R*^2^ = 0.6411, *P* < 0.01; Fig. 7k), and serum triglyceride (*R*^2^ = 0.7428, *P* < 0.01; Fig. 7l).

## Discussion

Using the obese mouse model with a high-fat diet and/or ovariectomy, this study showed that obesity increased adipose tissue with hypertriglyceridemia and insulin resistance. Notably, salubrinal attenuated obesity by reducing adipose tissue and corrected lipid and glucose metabolism. H&E, oil red O, and Masson’s trichrome staining showed that hepatic steatosis and fibrosis were present in the obese groups, whereas their pathological changes were suppressed by salubrinal. In vitro experiments showed that salubrinal significantly inhibited fatty degeneration in hepatocytes and reduced the formation of lipid droplets in adipocytes. Moreover, our study showed that salubrinal suppressed the ER stress by increasing p-eIF2α and ATF4 with a decrease in CHOP, and promoted autophagy by elevating LC3II/I with a reduction in p62. Correlation analysis showed that body fat was positively associated with body weight, hepatic adipocyte cell areas, and serum triglyceride, whereas the expression of p-eIF2α/eIF2α was negatively associated with body fat and lipid metabolism. Collectively, the result indicated that salubrinal alleviated hepatic steatosis and lipid deposition by inhibiting ER stress and altering autophagy in NAFLD.

NAFLD is a chronic pathological condition, characterized by lipid accumulation, which increases liver lipid levels, triglycerides, cholesterol, and free fatty acids^35,36^. Studies showed that hypercholesterolemia in response to ER stress leads to hepatic steatosis and alterations in hepatic lipid deposition^37,38^. Although hepatic steatosis is generally considered to be benign, it is a precursor of hepatitis, cirrhosis, and liver cancer^39^. With the progression of NAFLD, a disorder in lipid metabolism, often associated with post-menopause, elevates a risk of liver fibrosis with estrogen dificiency^4,5,40,41^. Consistently, our results showed that serum triglycerides and total cholesterol in the obese groups were significantly increased, suggesting a lipid metabolic disorder. Correlation analysis revealed that obesity-driven metabolic disorders could form a vicious circle and exacerbated pathological manifestations. Notably, salubrinal relieved the progression of hepatic steatosis and liver fibrosis and prevented NAFLD development.

Growing evidence suggests that ER stress is involved in the development of NAFLD by altering lipid synthesis^7,8,42,43^. In particular, eIF2α signaling has been shown to regulate lipogenesis and hepatic steatosis^10–13^. In this study, the presence of steatohepatitis in obese mice indicated that the ability to respond to the ER stress was impaired. The result showed that the increase in lipid deposition was accompanied by a significant decrease in the expression of p-eIF2α in hepatocytes and adipocytes. However, as an inhibitor of de-phosphorylation of elF2α, salubrinal altered the ER stress and alleviated lipid accumulation and hepatic steatosis. Correlation analysis also showed that the expression of p-eIF2α was negatively associated with lipid metabolism, and as a main linker between eIF2α signaling and apoptosis, CHOP was involved in lipid metabolism^43^. An animal study showed that inhibition of CHOP suppressed insulin resistance and reduced adipose tissue via eIF2α/CHOP signaling^44^. Consistent with this report, our result showed that CHOP was significantly increased in vivo and in vitro, while salubrinal inhibited its expression. However, this study did not focus on liver damage caused by OVX. In the future study, we plan to investigate the molecular mechanism of salubrinal on OVX-induced hepatocyte steatosis. Collectively, the result supports the notion that salubrinal regulated lipid metabolism and inhibited CHOP expression via eIF2α signaling.

A variety of studies indicate that autophagy, activated under the ER stress, was considered “endoplasmic stress-mediated autophagy”. Upregulation of autophagy under the ER stress alleviates hepatic steatosis and promoted lipid homeostasis in hepatocytes^15,45–47^. In vivo and in vitro experiments in this study showed that autophagy was inhibited when translational regulation in response to the ER stress was stimulated, while activation of autophagy was promoted in response to salubrinal. Of note, there are different reports on the regulation of autophagy to the ER stress. Some studies indicated the dual role of CHOP in the crosstalk between autophagy and apoptosis^48,49^. In other studies, autophagy was shown to be activated by PERK/eIF2α/ATF4 signaling as a protective response from apoptosis^50^. The latter view is consistent with our results, indicating that a high level of CHOP was significantly altered autophagy, whereas salubrinal partially reversed this alteration and inhibited CHOP. Although inhibition of the ER stress was beneficial to restore damaged cells, disruption of cytological stability caused by apoptosis may contribute to autophagy defects. Thus, salubrinal promoted autophagy through eIF2α signaling and decreased intracellular lipid accumulation, and alleviated hepatic steatosis.

**Fig. 8:**
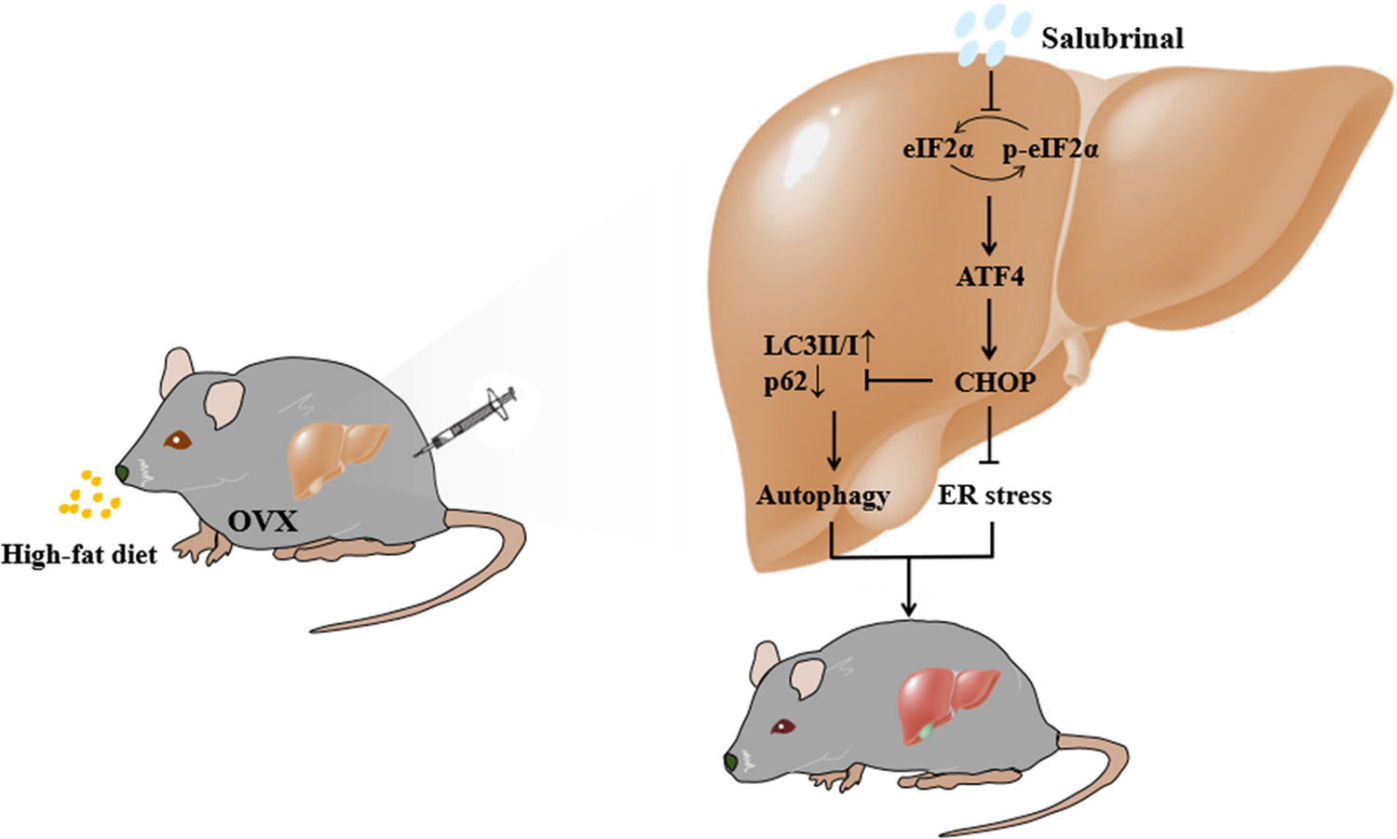
Mechanism of salubrinal’s action in hepatic steatosis. Salubrinal alleviated hepatic steatosis and lipidosis by inhibiting the ER stress and alerting autophagy through eIF2α signaling.

In summary, we demonstrated that eIF2α plays a critical role in the pathogenesis of obesity-induced NAFLD. The study herein utilized the obesity-induced mouse model as well as in vitro analysis of steatosis and ER stress and revealed that salubrinal was effective in attenuating fatty degeneration in the liver and inhibiting lipidosis. The present result also indicated a possible mechanism that salubrinal alleviated hepatic steatosis and lipidosis by inhibiting the ER stress and alerting autophagy through eIF2α signaling (Fig. 8). The current study supports the possibility of attenuating NAFLD through ER stress and autophagy-dependent lipid regulation.
